# Hormone PYY does not Explain the Variability in Weight Loss After Roux-en-Y Gastric Bypass: Evidence From a Meta-Analysis.

**DOI:** 10.1007/s11695-026-08559-8

**Published:** 2026-03-19

**Authors:** Giovanna Macanhã Scremin, Pedro Bicudo Bregion, Ana Cláudia Portes Nascimento, Júlia Marchiori Fein, Leonardo Halamy Pereira, Victor Kenzo Ivano, Everton Cazzo

**Affiliations:** 1 Pontifícia Universidade Católica do Paraná, Curitiba, Brazil; 2 State University of Campinas, Campinas, Brazil; 3 Clinics Hospital of Ribeirão Preto, Ribeirão Preto, Brazil; 4 Fluminense Federal University, Niterói, Brazil

**Keywords:** Peptide YY, Gastric bypass, Bariatric surgery, Weight loss, Incretins, Treatment failure

## Abstract

**Background:**

Peptide YY (PYY) plays a key role in postprandial satiety and is markedly increased after Roux-en-Y gastric bypass (RYGB). While GLP-1 has been widely studied, the role of PYY as a predictor of weight loss success remains unclear. Some patients are considered non-responders, achieving < 50% excess BMI loss (EBMIL) or experiencing significant weight regain. This systematic review and meta-analysis aimed to compare fasting PYY levels and postprandial area under the curve (AUC) between RYGB responders and non-responders.

**Methods:**

Following PRISMA guidelines, PubMed, Embase, and Cochrane databases were searched for observational or interventional studies assessing PYY in adult RYGB patients stratified by weight loss response. Primary outcomes were fasting PYY levels and PYY AUC after standardized meals. Random-effects meta-analysis was conducted using Review Manager 5.4. Mean differences (MD) with 95% confidence intervals (CI) were calculated. Heterogeneity was assessed via Cochran’s Q and I².

**Results:**

Five studies were included in the systematic review (92 non-responders and 89 responders), contributing to the fasting PYY meta-analysis, which showed no significant difference between groups (MD 1.71 pg/mL; 95% CI − 5.22 to 8.63; I² = 0%). Four studies reported AUC, also showing no difference (MD 53.16 pg·min/mL; 95% CI − 1726.29 to 1832.61; I² = 0%). Several studies observed lower satiety scores and attenuated appetite suppression in non-responders despite similar PYY profiles. No significant genetic variants in PYY or its receptor were associated with weight loss outcomes.

**Conclusion:**

RYGB responders and non-responders have comparable fasting and postprandial PYY concentrations. These findings suggest that differential weight loss after RYGB is likely mediated by factors beyond PYY secretion, including neural, behavioral, or other hormonal mechanisms.

## Introduction

Obesity remains a major public health crisis, with bariatric surgery representing the most effective intervention for sustained weight loss and comorbidity resolution. Among surgical options, RYGB [1] induces profound metabolic and hormonal changes, including elevated postprandial glucagon-like peptide-1 (GLP-1), peptide YY (PYY), and cholecystokinin (CCK), which enhance satiety and improve glycemic control [2].

Despite the overall success of RYGB, up to 50% of patients experienced primary weight loss failure (excess weight loss lower than 50%) or substantial weight regain [3]. The mechanisms underlying this variability are not fully understood. PYY, a gut-derived anorexigenic hormone secreted by L-cells in the distal gut, is markedly elevated post-RYGB and contributes to reduced appetite [4]. While PYY has been shown to predict glycemic outcomes, its predictive value for long-term weight loss remains controversial.

Previous studies [4–6] have yielded conflicting results, with some suggesting attenuated PYY responses in non-responders, and others showing no hormonal differences despite divergent weight loss. Clarifying this relationship is crucial to identifying patients at risk for suboptimal outcomes and to guiding personalized interventions. This systematic review and meta-analysis were conducted to quantitatively compare fasting and postprandial PYY levels in RYGB responders versus non-responders.

## Methods

### Protocol and Registration

This study followed PRISMA guidelines and was registered in PROSPERO (CRD420251126246).

## Search Strategy

We systematically searched PubMed, Embase, and Cochrane Library (inception to March 2025) for studies reporting PYY levels in RYGB patients classified by weight loss response. Search strategy terms were: *(bariatric OR “Roux” OR “gastric bypass” OR “RYGB” OR bariatric surgery OR “metabolic surgery”) AND (peptide YY OR “YY” OR “PYY” OR “PYY3-36” OR “hormones”)*. We manually reviewed the reference lists of previous meta-analyses and employed the snowballing technique to enhance our reference list.

## Study Selection

**Fig. 1:**
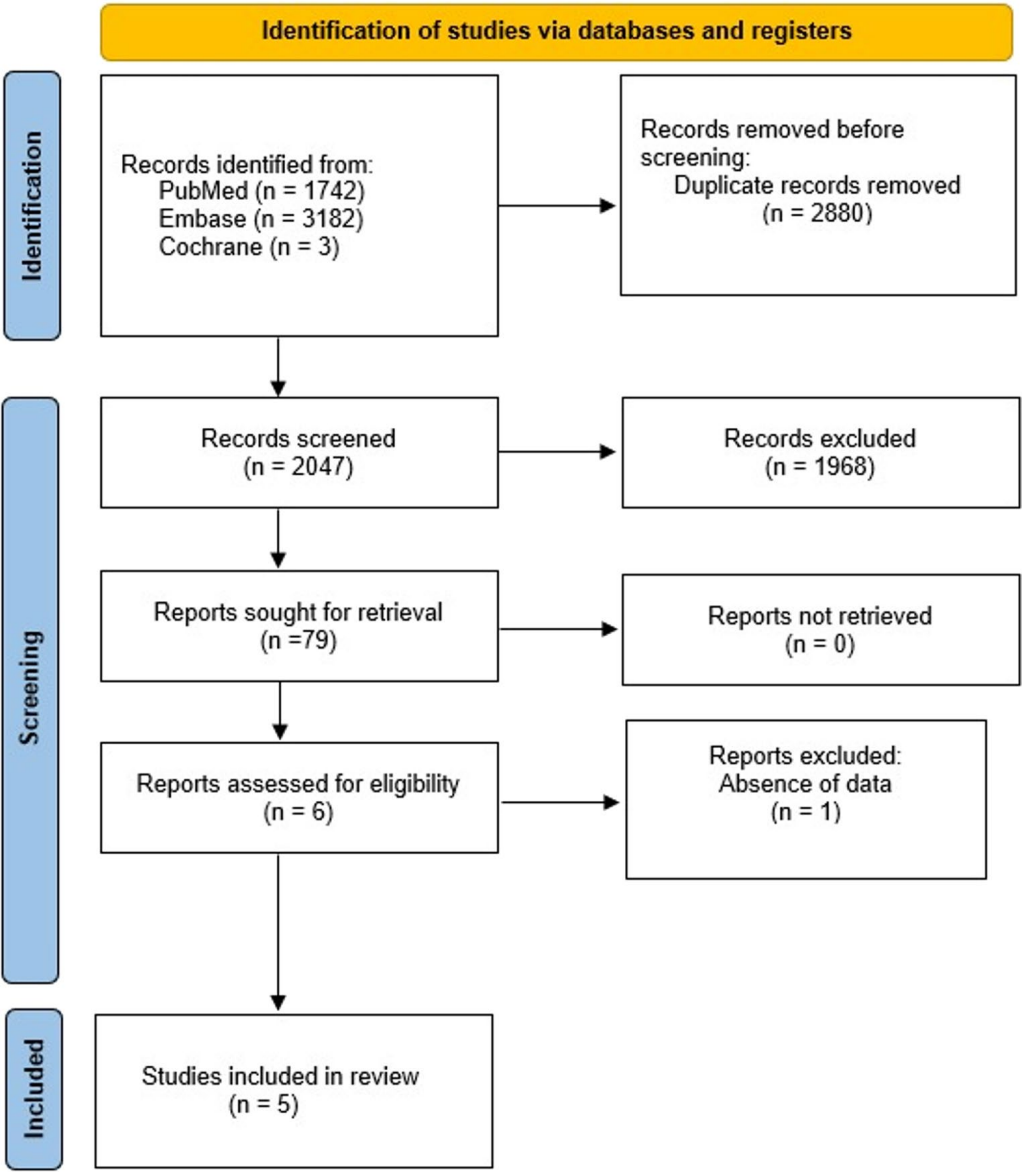
PRISMA 2020 flow diagram for screening and selected articles

The study selection was conducted in accordance with the Preferred Reporting Items for Systematic Reviews and Meta-Analyses (PRISMA) guidelines. Following the removal of duplicate records, two independent reviewers (GS and VK) conducted the initial screening. A third reviewer (EC) was consulted to resolve any conflicts. Titles and abstracts were assessed using predefined inclusion and exclusion criteria, and full-text versions of articles considered relevant were obtained for further evaluation (Fig. 1).

## Eligibility Criteria

Articles eligible for this review were observational studies or randomized trials written in English comparing PYY in RYGB patients, comparing weight loss responders vs. non-responders. PYY was assessed as fasting PYY and/or PYY area under the curve (AUC), depending on the methodology reported in the included studies.

Non-responders were defined as patients exhibiting primary insufficient weight loss, characterized by less than 50% excess weight loss (EWL), or as those meeting criteria for weight regain, according to the various definitions adopted across the included studies [7].

The exclusion criteria included non-English publications, studies without relevant outcomes for the review, conference abstracts and proceedings, case reports, non-comparative study designs, and studies that analyzed other gastrointestinal peptides.

Data extraction was carried out and reviewed by two independent authors (VK and GS) using Excel spreadsheets, with a third author (LP) verifying accuracy. The extracted variables included study characteristics (publication year, sample size on each group, study design, max follow-up and reported comorbidities) as well as patient demographics (age, sex and body mass index – BMI).

## Data Extraction and Quality Assessment

Two reviewers extracted data on study design, sample size, demographics, surgical details, follow-up time, test meal composition and caloric load, PYY assay methods, specific hormone forms (total vs. active), and outcomes. Data expressed as mean ± s.e.m (standard error of the mean) were converted to mean ± S.D. using the formula SD = S.E.M × √n. In the study by Lampropoulos et al. [8], the conversion from iAUC (incremental area under the curve) to AUC (area under the curve) was performed according to the formula described in the study’s own methods: iAUC = AUC – (90 × hormonal plasma concentration at baseline). Risk of bias was assessed using the ROBINS-I v2 for observational studies.

### Statistical Analysis

Continuous outcomes were pooled using inverse-variance random-effects models. Heterogeneity was quantified using I², with values > 50% considered substantial. Sensitivity analyses excluded outliers and high-bias studies. The AUC was analyzed using Review Manager 5.4. and WebPlotDigitizer. The standard deviation (SD) was calculated using the formula: (SD_AUC)² = (SD_iAUC)² + 90² × (SD_baseline PYY)². Furthermore, all measurements were converted and standardized to pg·mL⁻¹·min.

## Results

### Study Characteristics

Five studies met the inclusion criteria. Definitions of response varied, but most classified non-responders as < 50% EWL.

Non-responders were defined as patients presenting with insufficient primary weight loss (< 50% excess weight loss, EWL) or exhibiting weight regain, according to study-specific definitions. Responders were defined as those achieving satisfactory weight loss without significant regain during follow-up. Peptide YY (PYY) was evaluated as fasting PYY and/or PYY area under the curve (AUC) across studies. All studies used standardized mixed-meal tolerance tests (MMTT) for PYY AUC determination. Regarding the hormone forms, four studies [5, 6, 8, 11] measured Total PYY using ELISA or Multiplex assays, while one study [9] specifically quantified the active form (PYY 3–36) using radioimmunoassay (RIA). All studies utilized standardized mixed-meal tolerance tests (MMTT) or oral glucose tolerance tests (OGTT) for postprandial assessment.

A total of 183 patients from five observational studies were included, comprising 94 non-responders and 89 responders with complete demographic data. The pooled characteristics are summarized in Table 1.

**Table 1:** Main characteristics of the included studies

Study	Year	Number of patients (NRs/Rs)	Age (years)		BMI (kg/m²)		PYY isoform, assay method	Test meal composition and caloric intake in the first meal of the day
			Rs	NRs	Rs	NRs		
Bojsen-Møller et al.	2023	20/20	50.29 ± 3.74	50.59 ± 2.67	29.47 ± 0.77	39.50 ± 1.2	Total PYY, ELISA.	Fixed solid breakfast, 364 kcal
Dirksen et al.	2013	17/16	47,8 ± 2.1	48.2 ± 2.1	27.8 ± 0.9	38.9 ± 0.9	PYY3-36, RIA.	Solid mixed meal, 400 kcal
Lampropoulos et al.	2022	10/10	43.2 ± 6.1	44.6 ± 11.0	27.1 ± 3.5	33.0 ± 3.7	Total PYY, ELISA.	Standard liquid mixed meal (Fortimel Extra, Nutricia), 300 kcal
Nymo et al.	2024	25/25	52.5 ± 9.5	50.5 ± 6.3	27.0 ± 3.9	43.1 ± 5.7	Total PYY, LINCOplex.	Standardized liquid meal (Diben shake, Fresenius Kabi), 300 kcal
Sima et al.	2019	22/18	53.2 ± 11.4	52.0 ± 7.5	29.5 ± 3.5	40.6 ± 6.0	Total PYY, ELISA.	Oral Glucose Tolerance Test (75 g of glucose), 300 kcal

**Main characteristics of the included studies**: In Dirksen study the combined age was calculated from the means and standard deviations of each group using the weighted mean formula and the pooled standard deviation. In Lampropoulos study the age at follow-up was obtained by adding the mean age at surgery to the mean time since surgery, and the standard deviation was calculated as the square root of the sum of the squared standard deviations, assuming independence of the variables; NRs: non-responders; Rs: responders

Regarding the stimulation protocols, the caloric load of the test meals was consistent across the included studies, ranging from 300 to 400 kcal (see Table 1). Specifically, Lampropoulos et al., Nymo et al., and Sima et al. utilized a 300 kcal load (via liquid mixed meal or 75 g glucose), while Bojsen-Møller et al. and Dirksen et al. employed solid mixed meals containing approximately 364 kcal and 400 kcal, respectively.

Among non-responders, the mean age was 45.9 years (range 34.3–52.5; SD 7.4), with a predominance of females (81.2%). Mean BMI was 46.5 kg/m² (range 42.4–50.4; SD 3.0). The average time from surgery to study assessment was 4.3 years (range 1.6–8.9; SD 2.6). Basal metabolic rate (BMR) was reported in three studies, with a pooled mean of 1,518.2 kcal/day (SD 211.6).

Within responders, the mean age was 44.8 years (range 34.3–52.5; SD 7.3), with females comprising 80.5% of the sample. Mean BMI was 44.6 kg/m² (range 41.4–54.8; SD 3.7). The average time from surgery was 4.4 years (range 1.6–8.9; SD 2.7). BMR, reported in three studies, showed a pooled mean of 1,496.3 kcal/day (SD 201.3).

### Fasting PYY

**Fig. 2:**
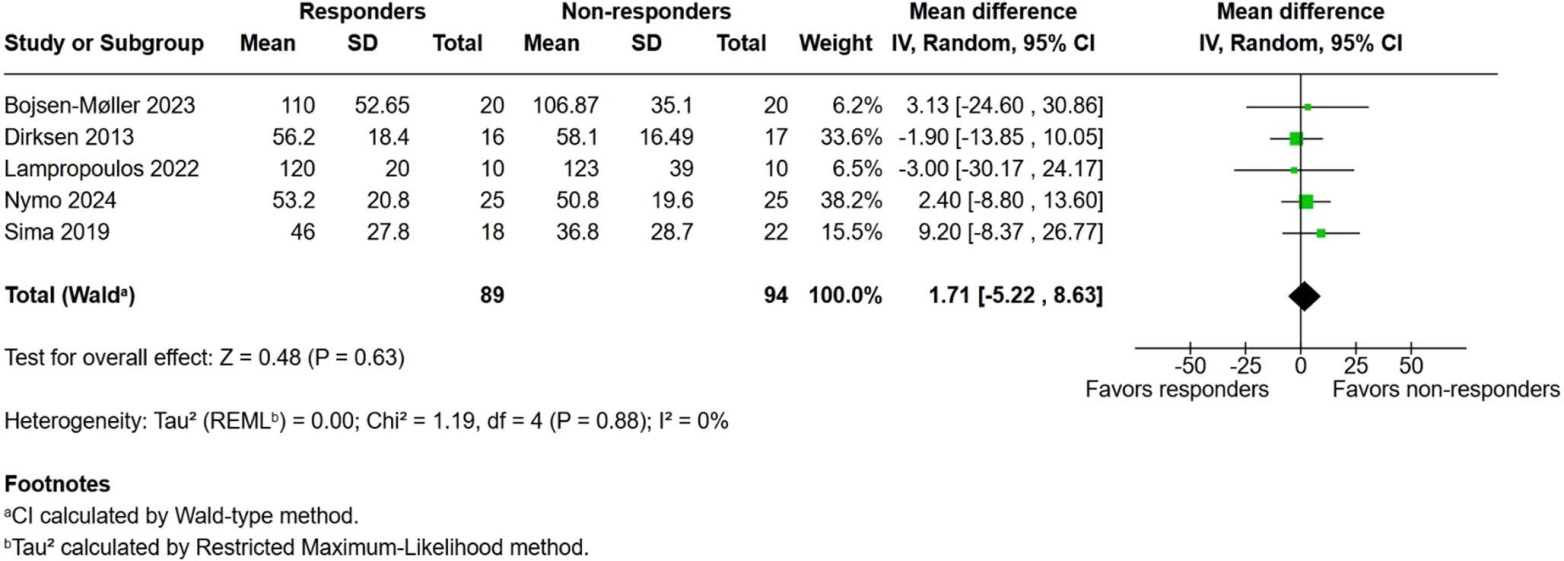
Fasting PYY levels

Regarding the measure of fasting PPY there was no significant difference between groups (MD 1.71 pg/mL; 95% CI − 5.22 to 8.63; I² = 0%; *p* = 0.63) (Fig. 2).

### Postprandial PYY AUC

**Fig. 3:**
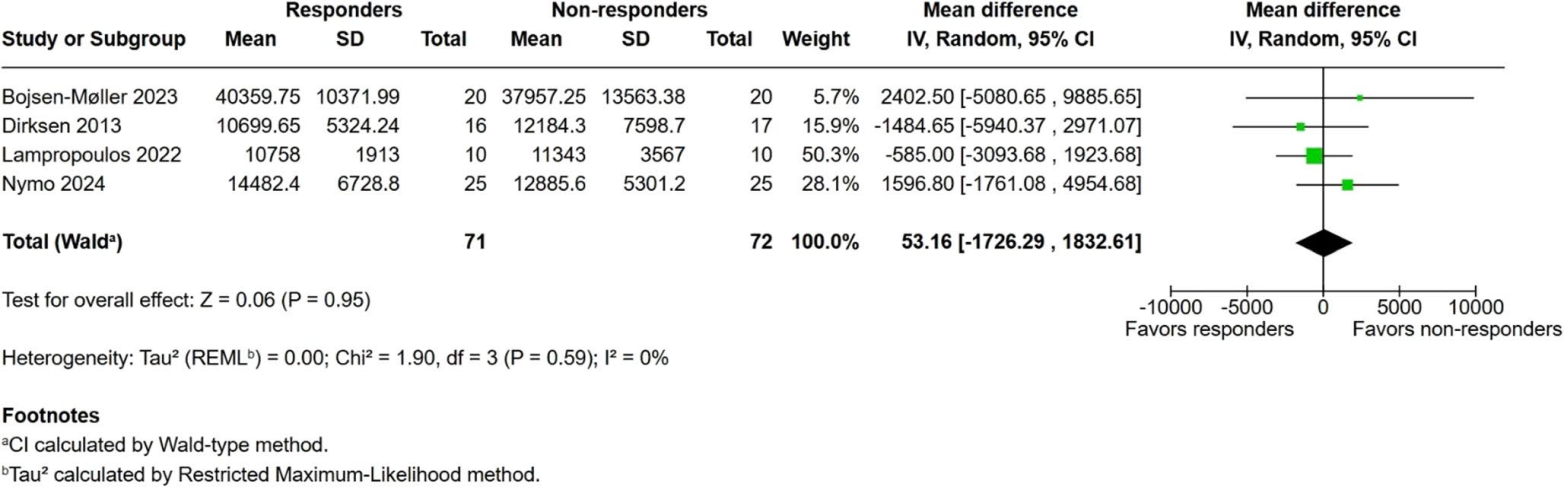
Postprandial PYY areas under the curve

Four studies reported PYY AUC after MMTT. No significant difference was found (MD 53.16 pg·min/mL; 95% CI − 1726.29 to 1832.61; I² = 0%; *p* = 0.95) (Fig. 3).

### GLP-1 and PYY

Bojsen-Møller et al. [5] reported similar fasting GLP-1 concentrations between non-responders and responders, with comparable postprandial responses after both standardized breakfast and ad libitum lunch. Under octreotide, postprandial GLP-1 tended to be higher in responders during the ad libitum meal. Dirksen et al. [9] found greater postprandial GLP-1 release in responders, whereas Nymo et al. [6] identified positive correlations between postprandial GLP-1 and PYY with total weight loss (%TWL) and excess weight loss (%EWL). Pereira et al. [10] and Sima et al. [11] found no significant differences in fasting or postprandial GLP-1 and PYY between groups but confirmed marked postprandial elevations in all participants.

### Ghrelin and CCK

In Bojsen-Møller et al. [5], fasting ghrelin was lower in non-responders, but postprandial suppression was similar between groups; octreotide reduced ghrelin similarly, with a trend toward greater reduction in those with suboptimal weight loss. Dirksen et al. [9] observed greater ghrelin suppression in responders, while non-responders showed lower fasting concentrations. Sima et al. [11] reported no fasting differences but noted that responders maintained ghrelin below baseline for longer. Regarding CCK, Bojsen-Møller et al. [5] found higher postprandial responses in non-responders during the ad libitum meal, consistent with Dirksen et al. [9], who also reported greater CCK release in poor responders.

### Other Gut Peptides

Pereira et al. [10] demonstrated that GIP and GLP-2 increased significantly postprandially in both groups, with correlations between GIP and lean mass, and negative correlations with fat percentage. Sima et al. [11] confirmed postprandial elevations in GIP without between-group differences.

### Leptin and Ghrelin/Leptin Ratio

Sima et al. [11] showed that fat mass–adjusted leptin was consistently higher in non-responders at fasting and throughout the oral glucose tolerance test, while responders had a higher fasting and early postprandial ghrelin/leptin ratio.

### Appetite Assessment

Bojsen-Møller et al. [5] found lower satiety and less hunger suppression after breakfast in non-responders. Dirksen et al. [9] reported a progressive reduction in pre-meal hunger (level of hunger just prior to food intake) across multiple meals only in RYGB responders. Nymo et al. [6] demonstrated that both fasting and postprandial desire-to-eat (DTE) and prospective food consumption (PFC) scores were significant predictors of %TWL and %EWL, independent of GLP-1 and PYY levels.

### Energy Expenditure and Metabolic Parameters

Dirksen et al. [9] reported higher resting energy expenditure (REE) in non-responders, although the difference disappeared after adjusting for body composition. Bojsen-Møller et al. [5] found no significant differences in basal energy expenditure or respiratory exchange ratio between groups. Minor differences in fasting glucose, C-peptide, and glucagon were observed, but postprandial responses were largely comparable.

### Pharmacological Modulation

Bojsen-Møller et al. [5] demonstrated that octreotide suppressed GLP-1, PYY, CCK, and ghrelin to a similar extent in both responders and non-responders, indicating preserved hormonal responsiveness to somatostatin analogues regardless of weight loss outcome.

### Technical Aspects

Across the included studies, the Roux-en-Y gastric bypass procedures demonstrated notable variability in technical aspects, which may influence hormonal and weight loss outcomes. In the study by Sima et al. [11], surgery consisted of creating a small gastric pouch with an alimentary limb length of 70 cm, and a biliopancreatic limb of 30 cm. Pereira et al. [10] reported performing a standard RYGB with a gastric pouch of 5 cm from the GE junction, a 150 cm alimentary limb, and a 100 cm biliopancreatic limb, without the use of a restrictive ring. In Nymo et al. [6], the gastric pouch volume was longer with 7 cm from the GE junction, anastomoses were constructed without a ring, and limb lengths were standardized at 100–150 cm for the alimentary limb depending on BMI, and a 40–60 cm for the biliopancreatic limb.

Lampropoulos et al. [8] described a distal bypass, with a 40 ml gastric pouch, a 400 cm alimentary limb, and a 100 cm common limb. Finally, in Dirksen et al. [9] and Bojsen-Møller et al. [5], gastric pouch and limb length were not reported.

### Risk of bias

**Fig. 4:**
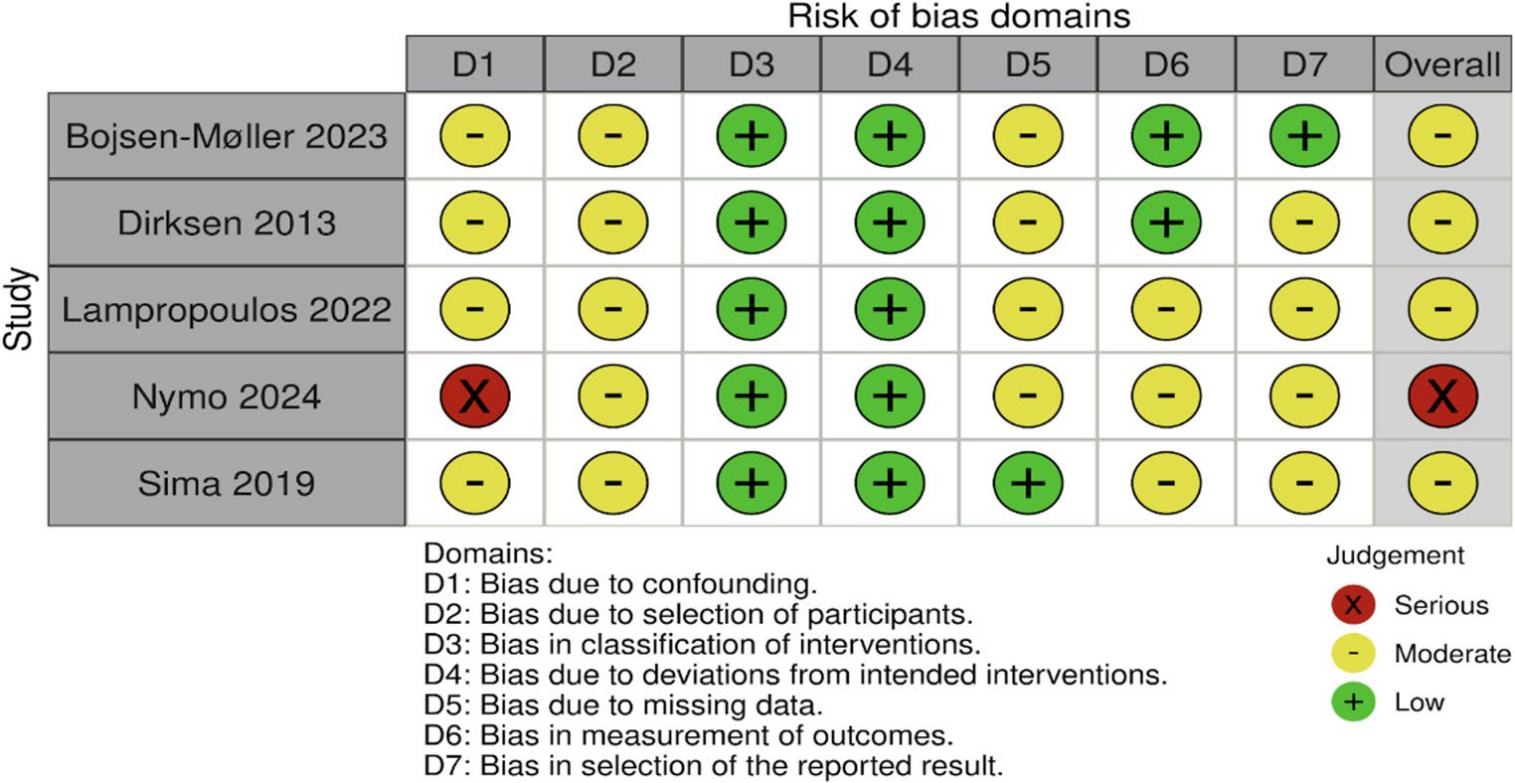
Risk of bias

In the five studies reviewed, the overall risk of bias ranged from moderate to serious. The domain most often driving these judgments was confounding, influenced not only by differences in current BMI, body composition, time since surgery, and medication use, but also by other unadjusted factors, including sex distribution, current age, history of diabetes or insulin resistance, habitual dietary intake and eating behaviors, physical activity levels, potential genetic or ethnic influences, use of nutritional supplements, and differences in nutritional status (e.g., deficiencies in iron, vitamin D, or protein). Participant selection presented a moderate risk: participants were typically identified from hospital records or earlier cohorts, and eligibility often depended on weight loss patterns and willingness to participate, both of which may introduce selection bias. Exposure or intervention classification was generally precise and low risk, as were deviations from intended interventions, given that the exposures were fixed. Moderate risk was more common in the domains of missing data and outcome measurement, often due to incomplete reporting of sample attrition or variability in hormonal assay performance. Selective reporting was considered low risk when prior trial registration was evident, but moderate otherwise, particularly when multiple exploratory outcomes were reported without a predefined analysis plan. Taken together, these findings indicate that the evidence base should be interpreted with caution. Figure 4 shows a graphic representation of the risk of bias analysis.

## Discussion

This meta-analysis demonstrates that both fasting and postprandial PYY concentrations are similar between RYGB responders and non-responders, suggesting that PYY alone does not explain interindividual variability in surgical weight loss. Mechanistically, this may imply that other satiety hormones (GLP-1, CCK) or gut-brain neural pathways may be more relevant in determining surgical outcomes; behavioral, dietary adherence, and psychological factors likely play a larger role in non-responder status; alterations in PYY sensitivity at the receptor or central nervous system level cannot be excluded.

These findings align with Bojsen-Møller et al. [5] and Dirksen et al. [9] who found similar gut hormone responses despite weight loss failure. Conversely, some interventional studies using somatostatin analogues (octreotide) showed that suppression of PYY and GLP-1 increases food intake in both groups, indicating functional relevance but not predictive capacity.

Insufficient weight loss (IWL) and weight regain (WR) are distinct but often overlapping clinical challenges following Roux-en-Y gastric bypass (RYGB) [9]. IWL is generally defined as an excess weight loss percentage (EWL%) of less than 50% at 18 months postoperatively, whereas WR refers to a progressive weight increase after an initially successful weight loss (EWL > 50%) [12]. Large multicenter and prospective studies illustrate the magnitude of the problem [13–15]. For example, the Swedish multicenter trial reported a 38% regain of maximal weight lost at 10 years after laparoscopic adjustable gastric banding (LAGB) and a 27.8% WR rate (range 14–37%) after laparoscopic sleeve gastrectomy (LSG) at ≥ 7 years follow-up [16]. In contrast, the Longitudinal Assessment of Bariatric Surgery (LABS) study found a smaller WR rate of 3.9% at 3–7 years after RYGB, suggesting a more durable weight loss profile for this technique [14].

The mechanisms underlying IWL and WR are multifactorial [17–21]. While increased caloric intake and maladaptive eating behaviors, such as grazing, are often observed in patients with suboptimal outcomes, these behaviors may be driven by underlying biological mechanisms rather than volitional non-adherence. For instance, although PYY secretion appears preserved, alterations in other homeostatic signals may drive energy intake. Sima et al. observed that non-responders exhibited higher fat-mass adjusted leptin levels and a more rapid return of ghrelin to baseline levels during glucose tolerance tests compared to responders. This hormonal profile suggests that non-responders may experience a stronger orexigenic drive and reduced satiety signaling from other pathways, making dietary modifications difficult to sustain without additional therapeutic support.

Furthermore, the issue may lie not in the secretion of gut hormones, but in the central processing of these signals. Bojsen-Møller et al. demonstrated that inhibiting gut hormones with octreotide increased food intake in responders but had no effect in non-responders, despite both groups having similar circulating hormone levels. This lack of behavioral response to hormonal manipulation suggests an impaired central anorectic response to gut signals in patients with primary weight loss failure. Consequently, therapeutic strategies should focus on overcoming this central resistance or targeting alternative pathways, rather than solely emphasizing behavioral compliance.

Physical inactivity also contributes to weight regain; however, barriers to exercise are often multifaceted, involving biomechanical limitations and psychological factors, rather than simple non-compliance. Mental health conditions, such as depression and binge eating disorder, are strongly associated with poor weight outcomes and require integrated management. Anatomical or technical surgical failures—such as pouch or gastrojejunostomy dilation, or gastrogastric fistula in RYGB—are additional contributors. Predictive factors for poor outcomes include older age, male sex, higher preoperative BMI, psychiatric comorbidities, and associated metabolic conditions such as T2DM, hypertension, and obstructive sleep apnea [17–21].

Prevention and treatment strategies for IWL and WR are multidimensional. Lifestyle reinforcement through dietary counseling, structured physical activity, and mental health support remains the foundation. Pharmacologic therapy, particularly GLP-1 receptor agonists, has emerged as an effective adjunct for patients with suboptimal weight loss after RYGB [22]. Endoscopic techniques—such as argon plasma coagulation, with or without transoral outlet reduction (TORe)—aim to reduce gastric pouch capacity and gastrojejunostomy diameter, though their availability is limited and long-term efficacy remains under investigation, with concerns about stricture formation and temporary effects [23–25]. Revisional surgical options are considered when less invasive strategies fail. Newer approaches, such as single-anastomosis duodenoileal bypass with sleeve (SADI-S) or single-anastomosis sleeve ileal bypass (SASI) [26–29], appear promising due to their potent incretin stimulation and relatively similar nutritional impact compared to distalization of RYGB, which often fails to elicit substantial additional weight loss, likely due to the persistent jejunal rather than ileal stimulation [30].

This meta-analysis has several limitations. First, all included studies were observational, carrying inherent biases. Second, definitions of non-responders and WR varied, and technical differences such as biliopancreatic limb length were not standardized. We attempted to minimize this by including only studies with matched control groups and by focusing exclusively on PYY responses after RYGB, avoiding the heterogeneity introduced by multiple surgical techniques or mixed hormonal endpoints. Another limitation concerns the test meals; although the caloric load was relatively similar across studies (300–400 kcal), the macronutrient composition of the test meals varied. While four studies [5, 6, 8, 9] used mixed meals containing fat and protein—potent stimulators of PYY secretion—one study (Sima et al.) utilized an Oral Glucose Tolerance Test (OGTT). This heterogeneity in nutrient composition represents a limitation, as the magnitude of the PYY response may differ between pure glucose and mixed-nutrient stimuli.

In conclusion, the findings suggest that PYY levels—both fasting and postprandial (AUC)—do not significantly differ between RYGB responders and non-responders, indicating that PYY may exert a smaller role in the pathophysiology of IWL and WR than other hormonal or behavioral factors. This reinforces the importance of a holistic management approach that addresses metabolic, anatomical, behavioral, and psychological domains to optimize and sustain weight loss after RYGB.

## Conclusion

Post-RYGB responders and non-responders exhibit comparable fasting and postprandial PYY levels. Differences in weight loss outcomes are likely mediated by other hormonal, neural, or behavioral mechanisms. Future research should integrate multimodal assessments of gut hormone function, eating behavior, and central appetite regulation to better predict surgical success.

## Data Availability

No datasets were generated or analysed during the current study.
